# Simultaneous triclosan degradation and nitrate reduction by a UV/Sulfite/Phenol process based on sulfite radical mechanism: dechlorination, mineralization, and bioassessment

**DOI:** 10.1371/journal.pone.0340396

**Published:** 2026-02-06

**Authors:** Hossein Faraji, Mehdi Salari, Forough Riahimanesh, Hadi Rahimzadeh, Behrouz Akbari-adergani, Asieh khalilpour, Nasim Naeeji, Fatemeh Asgharzadeh

**Affiliations:** 1 Tropical and Communicable Diseases Research Center, Iranshahr University of Medical Sciences, Iranshahr, Iran; 2 Noncommunicable Diseases Research Center, Sabzevar University of Medical Sciences, Sabzevar, Iran; 3 Department of Environmental Health Engineering, School of Public Health, Sabzevar University of Medical Sciences, Sabzevar, Iran; 4 Cellular and Molecular Research Center, Sabzevar University of Medical Sciences, Sabzevar, Iran; 5 Department of Environmental Health Engineering, Faculty of Health and Environmental Health Research Center, Golestan University of Medical Sciences, Gorgan, Iran; 6 Water Safety Research Center, Food and Drug Administration, Ministry of Health and Medical Education, Tehran, Iran; 7 Social Determinants of Health Research Center, Health Research Institute, Babol University of Medical Sciences, Babol, Iran; 8 Department of environmental health engineering school of public health, Babol university of medical science Babol, Iran; National Research and Innovation Agency, INDONESIA

## Abstract

Industrial development has increased the introduction of pollutants such as nitrate (NO₃⁻) and triclosan (TCS) into the environment, both of which pose significant environmental and health risks. The experiments were carried out in a 150 ml glass tube (photoreactor) equipped with a UV lamp (mercury vapor lamp (16 W) with a quartz cover operated at a controlled temperature (21 ± 2 °C), with an ultraviolet (UV) lamp and 2 mg/L dissolved oxygen. In the UV/sulfite process, the weak S-S bonds of sulfite, when exposed to UV radiation, produce SO3^•-^ radicals. The study assessed the effects of sulfite concentration, reaction time, pH, and pollutant levels on treatment efficiency. At pH 3 and 7 with 50 mg/L concentrations of TCS and NO₃ ⁻ , TCS removal efficiencies were 23% and 100%, while NO₃ ⁻ removal was 18% and 68%, respectively. Increasing sulfite from 50 to 200 mg/L improved results, achieving 85% NO₃⁻ and 100% TCS reduction in 90 minutes. SMP outperformed UV and sulfite-only treatments, achieving 68% and 100% reductions after 180 minutes. The process also led to partial nitrate-to-nitrite conversion, which decreased over time. In the SMP process, the values of Kobs (min-1) were 0.017, 0.031 for NO3- and TCS and EEM (kWh m-3), 0.3 and 0.2, respectively.Chemical oxygen demand (COD) and total organic carbon (TOC) reduced notably, and toxicity levels dropped dramatically. Given its high efficiency, low energy consumption, and minimal environmental impact, the SMP process shows significant potential for practical scale-up in wastewater treatment applications, especially in industries dealing with persistent organic pollutants and high nitrate concentrations.

## 1. Introduction

The contamination of water sources with organic pollutants such as triclosan (TCS) and inorganic compounds like nitrate (NO₃⁻) has been increasingly documented, primarily as a consequence of the discharge of untreated or insufficiently treated effluents from various sectors, including the chemical, petrochemical, and municipal industries [1,2]. Triclosan (TCS) or 5-chloro-2-(2,4-dichlorophenoxy) phenol with the chemical formula C_12_H_7_Cl_3_O_2_ and a molecular weight of 289.54 g/mol is a synthetic antimicrobial agent widely incorporated into personal and healthcare products, including shampoos, soaps, toothpastes, mouthwashes, cosmetics, and even treated textiles such as towels [1,3,4]. TCS is an organochlorine compound exhibiting physicochemical properties similar to those of phenolic compounds, mainly due to its chlorinated phenolic structure [5]. Excessive exposure to this substance can induce a range of adverse health effects, including dermatological disorders, endocrine dysfunction, carcinogenesis, and suppression of immune system function [6,7]. Triclosan (TCS) exhibits considerable environmental persistence and, under specific conditions such as exposure to ultraviolet (UV) light, it can undergo transformation into various dioxin derivatives. This chemical stability raises significant concerns regarding its long-term ecological impact [8,9].

Elevated concentrations of nitrate ions (NO₃⁻) in water can oxidize hemoglobin to methemoglobin, thereby impairing the blood’s oxygen-carrying capacity. This pathological condition, known as methemoglobinemia, can lead to severe hypoxia and, if left untreated, may ultimately result in death [10–12]. Biological denitrification processes are often associated with high operational costs and the generation of secondary water pollution, primarily due to the release of intermediate nitrogenous compounds. Consequently, an additional post-treatment step is required to effectively remove residual nitrogen species and ensure water quality standards are met [13]. Chemical technologies have shown considerable effectiveness in addressing environmental pollutants, primarily due to their capacity to degrade contaminants through the generation of active radical [14–16]. In general, advanced oxidation processes (AOPs) are characterized by radical production (OH^.^), while advanced reduction processes(ARP_S_) strongly produce reducing agents (e^-^_aq_) [17–20].

The advantages of this process over AOP include the high efficiency of the process in removing resistant halogen compounds, breaking C-Cl bonds in chlorinated organic compounds, reduction of the most stable state of nitrogen, ease of process control, and high reaction speed [21,22]. Additionally, regenerated materials, such as sulfite, can be activated in the presence of stimulatory agents, such as radiation, leading to the production of hydrated electrons, sulfite radical anions (SO₃^•-^), and hydrogen radicals (H^•^) [23]. sulfite (SO₃²⁻) is a specific anion used to describe a group of compounds, including sulfurous acid (H₂SO₃), bisulfite (HSO₃⁻), and sulfite (SO₃²⁻). The UV absorption spectrum of sulfite solutions is influenced by both the pH and the concentration of the solution [24,25] Exposure of sulfite solutions to UV radiation generates both oxidizing and reducing radicals. Reducing radicals, including hydrated electrons and the sulfite radical anion (SO₃•-), are formed in the solution. In advanced regeneration processes, sulfite reacts in the presence of a stimulating agent (e.g., radiation) to produce hydrated electrons, the sulfite radical anion (SO₃•-), and hydrogen radicals (H•), as described by reactions [1] and [2].


$${{\text{SO}}_{\text{3}}}^{\text{2}}+\;\text{hv}\;\to {{\text{SO}}_{\text{3}}}^{\cdot -}+\;{{\text{e}}^{-}}_{\text{aq}}$$ (1)

$${{\text{e}}^{-}}_{\text{aq}}\;+\;{\text{H}}^{+}\to {\text{H}}^{\cdot }$$ (2)

**Fig 1:**
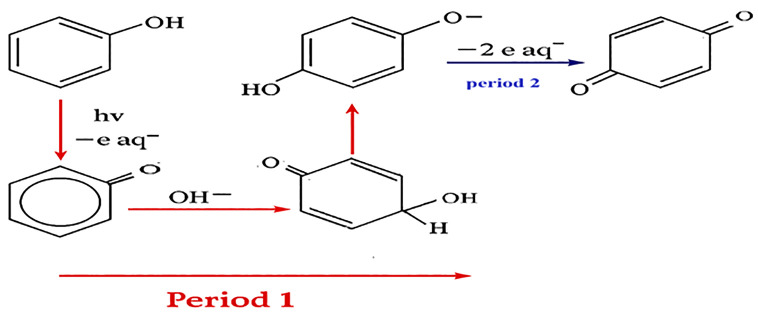
Electron generation from phenol in the presence of UV.

As illustrated in Fig 1, when phenolic compounds are exposed to UV radiation, they undergo transformation into p-benzoquinone, with p-hydroquinone acting as an intermediate compound. The formation of p-hydroquinone is attributed to the cleavage of carbon-oxygen dimers in the phenoxy radical (C₆H₅O^•^). This transformation occurs in two phases: in the first phase (Period I), p-hydroquinone is formed, while the second phase (Period II) is characterized by the conversion of p-hydroquinone into p-benzoquinone. Quantitatively, one mole of phenol releases one mole of electrons and one mole of phenoxy radicals, whereas one mole of p-hydroquinone releases two moles of electrons and one mole of p-benzoquinone. As shown in Fig 1, phenolic derivatives such as p-cresol, thymol, and (+)-catechin can be utilized for pollutant remediation [26]. In general, phenol acts as a photosensitizer or electron mediator that enhances the formation of SO₃•⁻ and eₐq and enhances electron transfer for pollutant degradation and nitrate reduction [14].

TCS and NO₃ ⁻ were selected as model contaminants due to their high environmental relevance and contrasting chemical characteristics. TCS is a widely used and persistent chlorinated organic micropollutant, whereas nitrate is a ubiquitous inorganic species commonly detected in wastewater. Their frequent co-occurrence and distinct reduction mechanisms provide an appropriate basis for assessing the simultaneous reductive removal of organic and inorganic contaminants by the SMP process.

This study presents an innovative approach that utilizes phenol, a compound structurally akin to TCS, as a UV-activated electron donor in combination with sulfite for enhanced pollutant degradation. While previous research has primarily focused on the individual removal of nitrate and organic pollutants through advanced oxidation or reduction processes (AOPs/ARPs), it was usually assumed in previous studies that ARP processes can better remove one type of pollutant. To address this gap, we introduce a UV/sulfite/phenol process capable of simultaneous TCS degradation and nitrate reduction. Therefore, the main objective of this study was to evaluate the effectiveness of the SMP (sulfite-phenol) process for the simultaneous removal of triclosan and nitrate in aqueous solutions, dechlorination, mineralization, and bioassay of TCS with Daphnia magna, and the percentage of nitrite, ammonium, and N_2_ selectivity, and to investigate the toxicity of the produced water. Assessing these three aspects individually is of particular importance: dechlorination of triclosan because it is an organochlorine compound that can persist in the environment; mineralization, which is crucial to ensure complete degradation of pollutants and avoid the accumulation of hazardous intermediates; and bioassessment, which is essential to guarantee that the final result is non-toxic.

## 2. Materials and methods

### 2.1. Materials and reagents

All chemicals were purchased from commercial suppliers (Sigma-Aldrich). Stock solutions of triclosan (TCS, 98%) and potassium nitrate (KNO₃, > 99%) were prepared separately by dissolving each compound in deionized water (resistance > 18 MΩ·cm). A 1000 mg/L stock solution was prepared for both TCS and KNO₃, and the solutions were stored at 4°C. Subsequently, standard solutions with concentrations ranging from 1 to 5 mg/L were prepared. The absorbance wavelengths for TCS and nitrate (NO₃⁻) were determined. The prepared standard solutions were injected into the instrument, and a calibration curve was constructed for each compound. These calibration curves were used to analyze all concentrations during the experiment.

### 2.2. Experience method

All experiments were conducted in model aqueous solutions. Triclosan and nitrate stock solutions were prepared using Milli-Q deionized water and diluted to the desired concentrations prior to use. All experimental procedures were conducted within a dedicated photoreactor and a custom-designed glass reactor chamber equipped with a quartz enclosure and a **UV-C** irradiation system. Depending on the reaction requirements, certain experiments were carried out under oxygen-free conditions, while in others, oxygen was supplied using an aeration pump to maintain the desired atmospheric composition. A 16 W mercury vapor UV lamp was housed within a quartz glass sleeve and positioned vertically at the center of the reactor chamber. To eliminate external light interference and enhance operational safety, the entire setup was enclosed with an aluminum shield.

**Fig 2:**
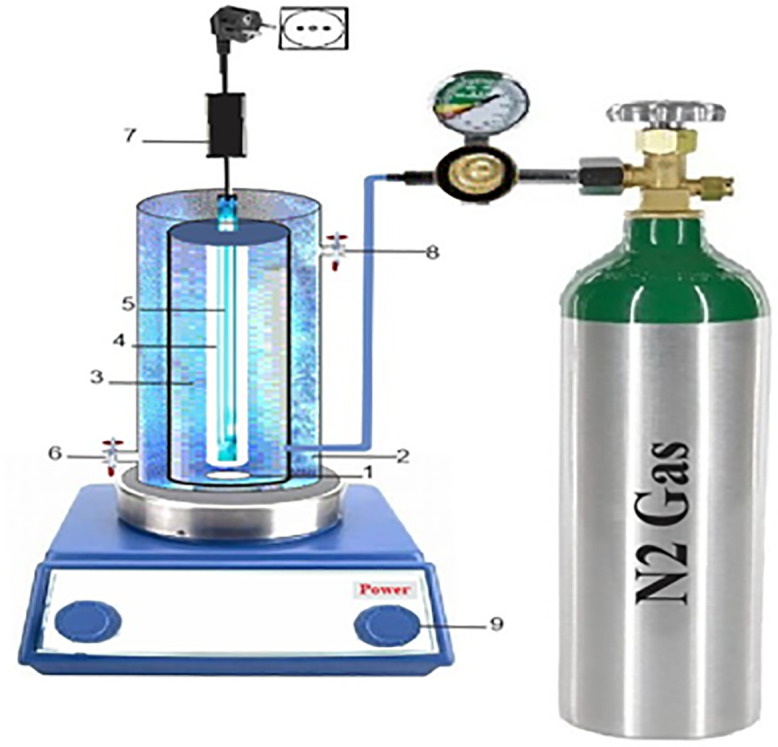
Photoreactor schematic: 1- Magnetic stirrer 2- Water jacket (with water circulation system) Photoreactor 3- Quartz Sleeve 4- Quartz tube 5- UV lamp 6- Inlet water flow 7- Power supply 8- Output water flow 9- Shaker.

A water jacket connected to a recirculating water bath was installed around the photoreactor to maintain a stable reaction temperature. Subsequently, the reactor was loaded with 150 mL of an aqueous solution containing predetermined concentrations of triclosan (TCS) and nitrate ions (NO₃⁻). The pH of the solution was adjusted to a predetermined value using NaOH (0.1) and sulfuric acid (0.1 normal) and then the required amount of sulfite was added to the reactor.and the system was subjected to UV irradiation. After mixing for a predefined period, aliquots of the solution were sampled and filtered through a 0.2 μm pore size Whatman membrane filter. Samples (5 mL) were withdrawn at regular intervals (0, 15, 30, 60, 90, 120, and 180 min) to monitor pollutant degradation and reduction kinetics. All experiments were conducted at a controlled temperature of 21 ± 2°C, under UV irradiation at an intensity of 95 mW cm ⁻ ², An aeration pump was used to provide oxygen, and the dissolved oxygen concentration was maintained at about 2 mg/L. The samples were analyzed for TCS and NO₃ ⁻ decomposition, mineralization efficiency, and regeneration behavior. Furthermore, the impact of radical scavengers and common water ions on the performance of the sulfite-mediated photoreduction (SMP) process was investigated under selected experimental conditions. Table 1. Shows the reaction conditions (variable and controlled parameters) in the experiment. In Fig 2. The experimental apparatus used in the present work, which is listed. During each experimental run, water samples were withdrawn from the batch reactor at predetermined time intervals for triclosan and nitrate analysis. Samples were immediately collected using clean glass syringes and prepared for subsequent chemical analyses. This study was approved by the Ethics Committee of Babol university of medical sciences (Code No: IR.MUBABOL.HRI.REC.1401.23).

**Table 1:** Reaction conditions (varied and controlled parameters).

parameter	pH	concentration (mg L ⁻ ¹)	Time(min)	DO(mg L ⁻ ¹)	UV intensity(mW cm ⁻ ²)
value	3, 7, 9	50, 100 150, 200	0, 15, 30, 60, 90, 120, 180	2	95

### 2.3. Kinetics of simultaneous reduction of NO_3_^-^ and TCS

In this study, C (mg L ⁻ ¹) denotes the concentration of either TCS or NO₃⁻ in solution, Crs (mg L ⁻ ¹) represents the concentration of free atoms or radicals, and k_obs_ (min ⁻ ¹) is the second-order observed rate constant. C₀ and C_t_ refer to the initial (inlet) and instantaneous (outlet) concentrations, respectively. The observed rate of degradation or reduction (robs, mg L ⁻ ¹ min ⁻ ¹) of TCS or NO₃⁻ during the SMP was determined based on kinetic models described by Equations (3–5).


$$\frac{𝐝{𝐂}_{𝐓𝐂𝐒\;𝐎𝐫\;𝐍𝐢𝐭𝐫𝐚𝐭𝐞}}{𝐝𝐭}=K{𝐂}_{𝐓𝐂𝐒\;𝐨𝐫\;𝐍𝐢𝐭𝐫𝐚𝐭𝐞}{𝐂}_{𝐫𝐬}$$ (3)

$${𝐂}_{𝐭}\;=\;{𝐂}_{0}e_{{\;-\;K}_{𝐨𝐛𝐬\;\;\;\;\;\;\;\;\;\;\;\;\;\;\;\;\;\;\;\;\;\;\;\;\;\;\;\;\;\;\;\;\;\;\;\;\;\;\;\;\;\;\;\;\;\;\;\;\;\;\;\;\;\;\;\;\;\;\;\;\;\;\;\;\;\;\;\;\;\;\;\;\;\;\;\;\;\;\;\;\;\;\;\;\;\;\;\;\;\;\;\;\;\;\;\;\;\;\;\;\;\;\;\;\;\;\;\;\;\;\;\;\;\;\;\;\;\;\;\;\;\;\;\;\;\;\;\;\;\;\;\;\;\;}}^{t}\;\;\;\;\;\;\;\;\;\;\;$$ (4)

$${𝐫}_{𝐨𝐛𝐬}\;=\;{-𝐤}_{𝐨𝐛𝐬}{𝐂}_{𝐓𝐂𝐒\;𝐨𝐫\;𝐍𝐢𝐭𝐫𝐚𝐭𝐞\;\;\;\;\;\;\;\;\;\;\;\;\;\;\;\;\;\;\;\;\;\;\;\;\;\;\;\;\;\;\;\;\;\;\;\;\;\;\;\;\;\;\;\;\;\;\;\;\;\;\;\;\;\;\;\;\;\;\;\;\;\;\;\;\;\;\;\;\;\;\;\;\;\;\;\;\;\;\;\;\;\;\;\;\;\;\;\;\;\;\;\;\;\;\;\;\;}$$ (5)

### 2.4. Analysis methods and performance indicators

To determine the concentration of triclosan (TCS) in the samples, high-performance liquid chromatography (HPLC) analysis was conducted using a CECILCE 4100 Knauer instrument equipped with a C18 column (250 mm × 4.6 mm, 5 μm particle size). The chromatographic separation was carried out at a constant column temperature of 30 °C, with a mobile phase consisting of acetonitrile and water (85:15, v/v) delivered at a flow rate of 1.0 mL/min. Prior to injection, all sample and standard solutions were filtered through 0.2 - + μm syringe filters (MDI, India) to remove particulate matter and prevent column clogging. Where necessary, samples were diluted appropriately to ensure analyte concentrations remained within the linear range of the calibration curve.

To identify intermediate transformation products generated during the reaction, HPLC-MS analysis was performed using an API2000 mass spectrometry system (AB SCIEX) [27]. The extent of mineralization was evaluated by measuring total organic carbon (TOC) using a TOC analyzer. Chemical oxygen demand (COD) was assessed using the closed reflux method with potassium dichromate, following Standard Method 5220 B [28]. Ammonia nitrogen levels were quantified via the Nessler method, in which the intensity of the yellow coloration produced is directly proportional to the ammonia concentration. Absorbance was measured at 425 nm using a DR 5000 spectrophotometer (HACH, USA), ensuring reliable detection in aqueous samples [29]. Concentrations of nitrate and nitrite were also determined spectrophotometrically in accordance with the procedures described in the 23rd edition of *Standard Methods for the Examination of Water and Wastewater*. All absorbance measurements were performed using the same DR 5000 spectrophotometer to maintain consistency and analytical precision throughout the study [30].

Eq. (6) and (7) were used to account fo percentage of TCS degradation and mineralization in the SMP process.


$$\text{TCS}\;\;\text{degradation}\;\;(\%)=\;\frac{\left({\text{TCS}}_{\text{in}}-{\text{TCS}}_{\text{out}}\right)}{{\text{TCL}}_{\text{in}}}\;\times \text{100}\;$$ (6)

$$\text{TOC}\;\;\text{mineralization}=\frac{\left({\text{TOC}}_{\text{in}}-{\text{TOC}}_{\text{out}}\right)}{{\text{TOC}}_{\text{in}}}\;\times \text{100}\;$$ (7)

where TCS represents the concentration of TCS at the input and output of the SMP process. Respectively. Also, TOC_in_ and TOC_out_ demonstrate the initial and final concentration of TOC in the aqueous solution [31].

To calculate the percentage of dechlorination, the following equation was used, where C_Cl-_ and C_0_ are the amount of released chlorine (in mol L^-1^) in the solution and the concentration of TCS solution (mol L^-1^), respectively [21] Eq. (8).


$$\text{Dechlorination}\;\text{percentage}=\frac{{\text{C}}_{{\text{cl}}^{-}}}{{\text{C}}_{\text{0}}\times \text{3}}\times \text{100}$$ (8)

Where C_Cl⁻_ represents concentration of released chloride ions (mol/L), and C _₀_ denotes initial concentration of triclosan (mol/L). The factor 3 in the equation accounts for the number of chlorine atoms present in a single TCS molecule.

Transformation products formed during the SMP process were investigated by HPLC–MS using an API2000 (AB Sciex). Identification of intermediates was based solely on their observed m/z values and retention times. All detected species, along with their m/z, retention time and proposed formulas, are listed in Supplementary S1 Table in (S1 File), and the corresponding spectra are provided in Supplementary S1 File. Due to the unavailability of certified standards for these intermediates, the analysis was limited to qualitative identification based on m/z values and retention times.

### 2.5. Performance of the reduction process for nitrate reduction

Under nitrate reduction conditions, various intermediates and final products such as ammonium (NH₄⁺), nitrite (NO₂⁻), total organic nitrogen (TON), nitric oxide (NO), nitrous oxide (N₂O), and nitrogen gas (N₂) are formed.

The selectivity percentage reflects the extent to which nitrate is converted into specific products during the reduction process. Specifically, selectivity to nitrite and total organic nitrogen indicates the proportion of nitrate transformed into these species.

The performance of the nitrate reduction process was evaluated based on the overall nitrate removal efficiency (Equation (9)), the selectivity toward nitrite formation (Equation (10)), the selectivity toward ammonium production (Equation (11)), and the selectivity toward nitrogen gas generation (Equation (12)) [32].


$$\text{Nitrate}\;\text{reduction}(\%)=\frac{\left({{{{\text{NO}}_{\text{3}}}^{-}}_{\text{in}\;}}_{-}\;{{{\text{NO}}_{\text{3}}}^{-}}_{\text{out}}\right)}{{{{\text{NO}}_{\text{3}}}^{-}}_{\text{in}}}\;\times \text{100}\;$$ (9)

$$\text{Nitrite}\;\text{selectivity}(\%)=\;\frac{{{{\text{NO}}_{\text{2}}}^{-}}_{\text{out}}}{{{{\text{NO}}_{\text{3}}}^{-}}_{\text{in}}\;\_\;{{{\text{NO}}_{\text{3}}}^{-}}_{\text{out}}}\times \text{100}$$ (10)

$$\text{Ammonium}\;\text{selectivity}(\%)=\;\frac{{{{\text{NH}}_{\text{4}}}^{+}}_{\text{out}}}{{{{\text{NO}}_{\text{3}}}^{-}}_{\text{in}\;}\_\;{{{\text{NO}}_{\text{3}}}^{-}}_{\text{out}}}\times \text{100}$$ (11)

$$N_{\text{2}}\;\text{selectivity}(\%)=\;\frac{{{{\text{NO}}_{\text{3}}}^{-}}_{\text{in}-[{{{(\text{NO}}_{\text{3}}}^{-}}_{\text{out}})+({{{\text{NO}}_{\text{2}}}^{-}}_{\text{out}})+({{{\text{NH}}_{\text{3}}}^{+}}_{\text{out}})]}}{{{{\text{NO}}_{\text{3}}}^{-}}_{\text{in}\;}\_\;{{{\text{NO}}_{\text{3}}}^{-}}_{\text{out}}}\times \text{1}\text{0}\text{0}$$ (12)

Where NO_3_^-^ in and NO_3_^-^ outrepresent the influent and effluent concentrations of nitrate (mol/L), respectively, before and after the treatment process.

### 2.6. Determining the toxicity using *Daphnia Magna*

The effectiveness of the SMP process in reducing effluent toxicity under alternating flow conditions was assessed. Raw water samples with an initial TCS concentration of 50 mg L ⁻ ¹ were prepared. For the SMP process, the inlet conditions were set as follows: TCS concentration of 50 mg L ⁻ ¹, sulfite concentration of 100 mg L ⁻ ¹, pH adjusted to 7, reaction time of 90 minutes, UV intensity of 95 mW cm ⁻ ², dissolved oxygen concentration of 2 mg L ⁻ ¹, and temperature maintained at 21 ± 2 °C. All experiments were conducted under natural light conditions.

Treated wastewater samples were diluted to final concentrations of 100%, 75%, 50%, 25%, 12%, 6%, 3%, 1%, and 0.5% (control), each prepared in 100 mL volumes. Ten neonate *Daphnia magna* were introduced into each dilution. The number of live and dead daphnids was recorded after 24, 48, 72, and 96 hours of exposure. The *Daphnia magna* used in this study were sourced from the Caviar Fish Breeding Center of Mazandaran. In addition to being more sensitive to environmental pollutants, Daphnia magna has a special place in environmental experiments today due to its short reproductive period, high sensitivity, simplicity of the test, and low cost, as well as its monogamy and the production of genetically identical offspring of the same sex [33].

Toxicity was quantified based on mortality rates, and ${LC}_{\text{5}\text{0}}$ values were calculated using the probit analysis method in SPSS software. In addition, the toxicity unit (TU) of the effluent was determined according to Equation [34].


$$\text{TU}=\frac{\text{100}}{\text{LC50}}\;\;$$ (13)

### 2.7. Electrical energy consumption and total system cost

The electrical energy consumption (EEM, kWh m ⁻ ^3^) was calculated using Equation (14), where *P* represents the electrical power input (kW), *V* is the volume of the solution treated inside the reactor (m^3^), *t* is the reaction time (h), and *C₀* and *Cₜ* denote the initial and final concentrations (mg L ⁻ ¹) of either triclosan (TCS) or nitrate, respectively.

This calculation allows for the evaluation of the specific energy demand associated with the degradation or reduction processes. Furthermore, the total operational cost of the system can be estimated based on the energy consumption and the prevailing electricity price [35].


$$E_{EM}=\left[\;\;[\frac{P\times t}{V\times log\left(\frac{C_{O}}{C_{t}}\right)}!\;\right]\;$$ (14)

$$\text{total}\;\text{system}\;\text{cost}=\text{1}.\text{4}\text{5}\times E_{EM}\times Power\;cost\;\times reductant\;cost$$ (15)

According to Equation (14), *P* represents the power of the UV lamp (kW), *t* is the irradiation time (minutes), *V* is the volume of the treated solution (L), and *C₀* and *Cₜ* are the initial and final concentrations of the pollutant (mg L ⁻ ¹) [36]. Equation (15) is also used to calculate the total system cost (TSC, $ m ⁻ ^3^), where the energy consumption (E_EM_) is expressed in kWh m ⁻ ^3^, the electricity cost is given in $/kWh, and the reductant cost is expressed in $/g [36].

## 3. Findings and discussion

### 3.1. Effect of solution pH on SMP process in TCS and NO_3_^–^ reduction

**Fig 3:**
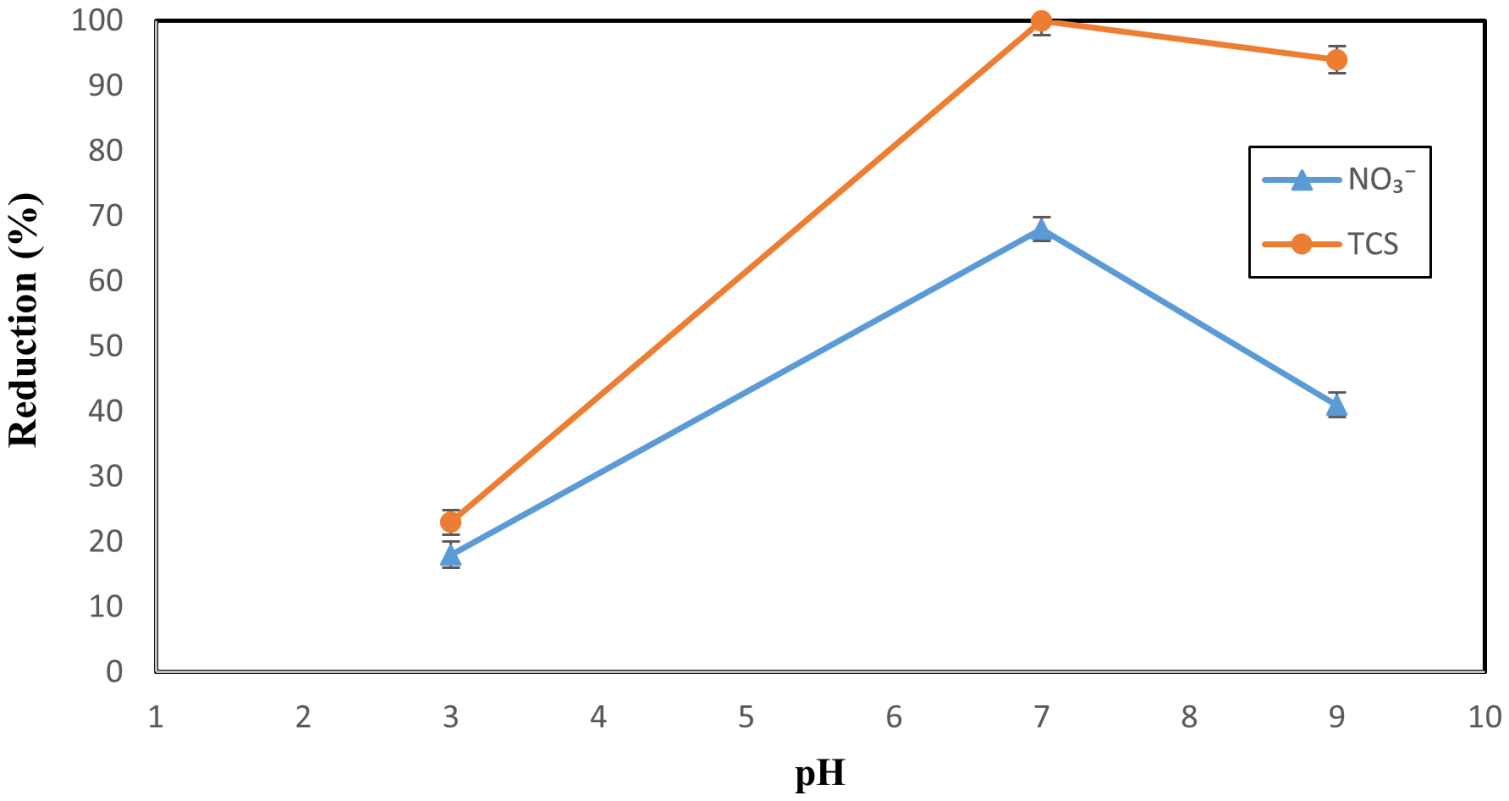
Effect of the pH on the nitrate and TCS reduction with SMP process (sulfite  = concentration, 100 mg L^-1^; TCS = 50 mg L^-1^ and nitrate = 50 mg L^-1^).

The influence of pH [3,7,9] on the reduction efficiency of triclosan (TCS) and nitrate (NO₃⁻) using the SMP process was investigated. In this study, the concentration of NO₃ ⁻ was set at 50 mg L ⁻ ¹, TCS at 50 mg L ⁻ ¹, and sulfite concentration at 100 mg L ⁻ ¹, with a reaction time of 90 minutes. As shown in Fig 3, the reduction efficiencies at pH 3 and 7 were 18% and 68% for NO₃ ⁻ , and 23% and 100% for TCS, respectively. The pH distribution and the relative concentration of the pollutants were influenced by high sulfite concentrations, including H₂SO₃, HSO₃ ⁻ , and SO₃^2^ ⁻ , which enhanced the reduction process through the formation of reactive intermediates [37,38]. The behavior of sulfite species, such as bisulfite (HSO₃⁻) and sulfite (SO₃^2^⁻), under acidic and alkaline conditions, exhibits distinct UV absorption properties that are pH-dependent. These species show different absorption spectra at various wavelengths, which can be attributed to the unique UV absorption characteristics of bisulfite and sulfite. This wavelength-dependent UV absorption leads to the generation of reactive radicals, including H•, hydrated electrons (eₐq⁻), and sulfite radicals (SO₃•⁻) [17,28]. The formation of these radicals plays a crucial role in the reduction process [24,39]. Bisulfite exhibits strong UV absorption at 254 nm, whereas sulfite displays relatively limited absorption across the 225–300 nm range. This difference in absorption behavior significantly influences the generation of reactive species under UV irradiation, thereby affecting the efficiency of reduction processes [37]. This absorption behavior indicates that bisulfite, when exposed to irradiation at 254 nm, exhibits limited photochemical activity and is unable to effectively generate hydrated electrons and free radicals. This limitation highlights the need for either alternative activation pathways or the presence of coexisting species to enhance radical generation under these conditions [40]. Moreover, the scavenging of hydrated electrons by protons (H⁺) under acidic conditions must be carefully considered. At lower pH values, where the formation of hydrogen radicals (H•) is favored, hydrated electrons and other reactive species play a crucial role in facilitating halogenation reactions. This interplay between radical generation and proton-mediated electron consumption significantly influences the overall reactivity of the system [41]. According to previous studies conducted by some researchers, the UV absorption of different species of reductant is different and pH-dependent [42]. These results show that SMP operates optimally under near-neutral conditions due to balanced radical production and minimal electron absorption, indicating potential applicability to real wastewaters with moderate pH [21]. In the study by Dadrick et al., the results showed that nitrate reduction was not affected under acidic pH conditions (2.6) in a photocatalytic process with TiO2/Ag [43].

### 3.2. Effect of Sulfite Concentration on the SMP Process for TCS and NO₃ ⁻ Reduction

**Fig 4:**
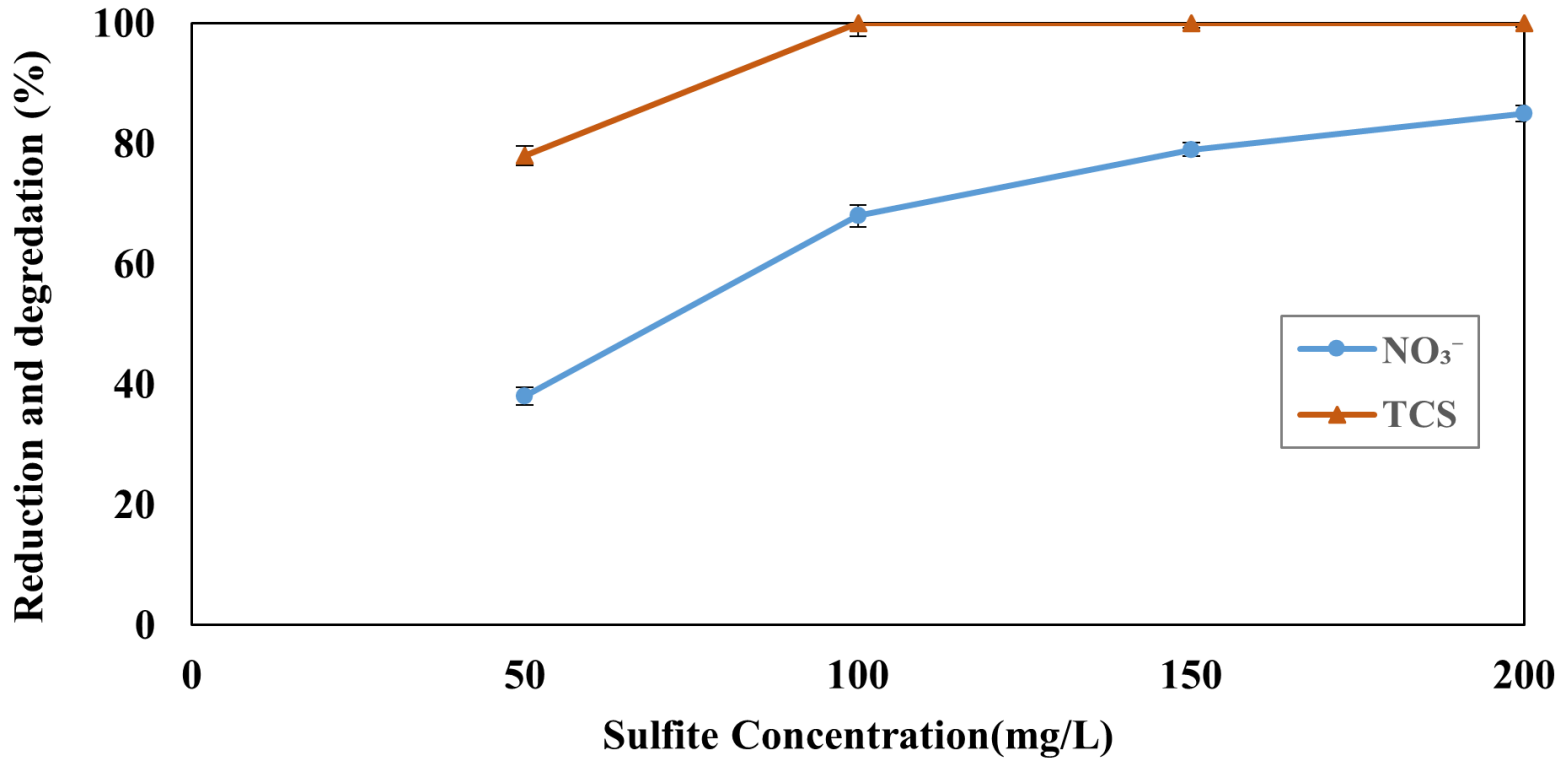
Effect of sulfite concentration on the nitrate and TCS reduction (pH, 7.0, Time, 90 min, TCS  = 50 mg L^-1^ and nitrate = 50 mg L^-1^).

The influence of sulfite concentration on the reduction efficiency of TCS and NO₃⁻ by the SMP process was systematically investigated. In these experiments, initial concentrations of both NO₃⁻ and TCS were maintained at 50 mg L ⁻ ¹, and the reaction time was fixed at 90 minutes. As shown in Fig 4, an increase in sulfite concentration from 50 to 200 mg L ⁻ ¹ significantly enhanced the removal efficiencies. Specifically, the reduction of NO₃⁻ and TCS improved from 38% and 78% at 50 mg L ⁻ ¹ sulfite to 85% and 100% at 200 mg L ⁻ ¹ sulfite, respectively. This trend demonstrates that higher sulfite dosages enhance the generation of reactive species, such as hydrated electrons and sulfite radicals, thereby accelerating the reduction reactions. Consequently, the enhanced sulfite concentration under otherwise identical experimental conditions led to a higher reaction rate and improved overall process performance [44]. According to the Beer–Lambert law, an increase in sulfite concentration enhances UV absorption, thereby promoting the photolysis of sulfite ions. This, in turn, leads to the generation of a higher concentration of reactive radicals, which are primarily responsible for the degradation of TCS. Therefore, the elevated sulfite concentration improves TCS decomposition efficiency by increasing both the extent of radiation absorption and the availability of reactive species necessary for effective pollutant breakdown [45]. The enhanced removal efficiency of TCS and NO₃⁻ with increasing sulfite concentration observed in this study is mechanistically supported by prior work on UV/sulfite-based advanced reduction processes. In particular, Sheikhmohammadi et al. (2025) demonstrated that increasing sulfite concentration in the UV/trichlorophenol/sulfite (UV/TCP/S) system directly amplified the generation of reactive species, such as hydrated electrons (e ⁻ ₐq), sulfate radicals (SO₄ ⁻ •), and phenoxy radicals (PhO•). These species played a pivotal role in the reductive transformation of Cr(VI) to Cr(III). A higher sulfite dose enhanced UV photon absorption, thus intensifying the photolysis of sulfite and leading to more abundant formation of reactive radicals. Importantly, the study also highlighted that excessive sulfite can act as a scavenger of radicals under certain conditions, potentially inhibiting the process if not optimized. Therefore, consistent with the present findings, increasing sulfite concentration up to an optimal level significantly boosts radical generation, promoting more efficient degradation of pollutants such as TCS and NO₃⁻ via the SMP mechanism [46]. The synergistic effect of optimal pH and sulfite concentration shows that the efficiency of radical formation is maximized under conditions of moderate proton availability, while excessive acidity suppresses the stability of the hydrated electron [47].

### 3.3. Comparison of the efficiency of UV, Sulfite and SMP processes in reduction of NO_3_^-^ and TCS

The performance of UV irradiation, sulfite addition, and the combined sulfite-mediated photoreduction (SMP) process in the simultaneous reduction of NO₃⁻ and TCS from aqueous solutions was evaluated, and the results are presented in Supplementary S1 Fig in (S1 File). The reduction efficiencies for NO₃⁻ and TCS were 18% and 21% for the sulfite process, 24% and 67% for the UV process, and 68% and 100% for the SMP process, respectively. The results also show that TCS degradation and nitrate reduction were achieved in the separate method at 100% and 60%, respectively. However, under simultaneous conditions, TCS degradation was slightly reduced in the presence of nitrate, such that the TCS efficiency decreased to 95%. However, nitrate reduction was enhanced in the presence of TCS, such that the nitrate reduction efficiency increased to 68%.

The relatively low efficiencies observed with UV or sulfite alone can be attributed to the limited generation of reactive radicals (e.g., SO₃^•⁻^) and hydrated electrons under these conditions. The markedly superior performance of the SMP process compared to UV or sulfite alone is consistent with findings in other advanced oxidation/reduction systems. For instance, Pratiwi et al. (2024) reported that the UV/H₂O₂ process achieved a significantly higher degradation efficiency (62.43%) for amoxicillin than UV alone (30.52%) or H₂O₂ alone (20.36%) under similar operational conditions. This enhancement was attributed to the synergistic production of hydroxyl radicals (OH^•^) when UV light activates H₂O₂, which substantially increases the oxidative potential of the system. Analogously, in the present SMP process, the combination of UV and sulfite promotes the simultaneous generation of hydrated electrons and sulfite radicals—species that are highly effective for both reductive and oxidative degradation pathways. These results collectively underscore the crucial role of synergistic radical generation in hybrid processes for the efficient removal of persistent organic and inorganic contaminants such as TCS and nitrate [48]. Furthermore, the low absorption cross-section of TCS and NO₃⁻ under UV irradiation further limits their direct photodegradation. In contrast, the SMP process demonstrated markedly enhanced performance, which is primarily due to the synergistic generation of high concentrations of reactive radicals and hydrated electrons, making it a highly effective method for the concurrent reduction of NO₃⁻ and TCS [37,49].

### 3.4. Kinetics studies of NO_3_^-^ and TCS regeneration reaction

Table 2 presents the kinetic parameters determined for the degradation of TCS and the reduction of NO₃⁻ under different treatment conditions. The results demonstrate that the SMP process exhibited superior kinetic performance compared to UV irradiation alone and sulfite addition alone. Specifically, the observed rate constants (K obs, min ⁻ ¹) and observed reaction rates (r_obs, mg L ⁻ ¹ min ⁻ ¹) for TCS degradation were 0.030 and 1.50 for the SMP process, 0.024 and 1.12 for UV alone, and 0.001 and 0.05 for sulfite alone, respectively. Similarly, for NO₃ ⁻ reduction, the corresponding K_obs_ and r_obs_ values were 0.015 and 0.89 for SMP, 0.003 and 0.24 for UV alone, and 0.001 and 0.05 for sulfite alone. These findings clearly indicate that the synergistic effects of sulfite and UV in the SMP process significantly enhance the generation of reactive species, resulting in markedly higher reaction rates for both TCS and NO₃ ⁻ compared to the individual processes. In the photocatalytic study, it was found that N-F/TiO2 was effective in removing pentachlorophenol with a degradation rate of 99% and a value of (kobs) of 0.005 (min ^−1^) [50].

**Table 2:** Kinetics of NO_3_^-^ and TCS regeneration reaction.

Process	R ^2^		K_obs_(min ^- 1^)		r_obs_(mg L^- 1^ .min)	
	NO_3_^-^	TCS	NO_3_^-^	TCS	NO_3_^-^	TCS
alone UV	0.92	0.96	0.003	0.024	0.24	1.12
Sulfite alone	0.91	0.96	0.001	0.001	0.05	0.05
SMP	0.99	0.98	0.015	0.03	0.89	1.5

### 3.5. Evolution of Ammonium, Nitrite, Nitrogen Gas, and pH During the Reduction Process

**Fig 5:**
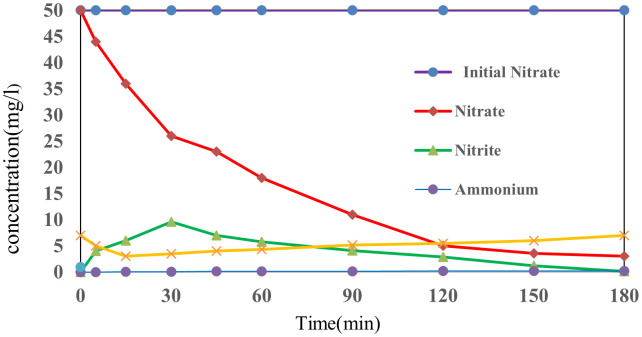
Concentration of ammonium, nitrite, and nitrate, Initial nitrate = 50 mg L^-1^,Initial pH = 7, Sulfite = 150 mg L^-1^.

During the reduction of nitrate (NO₃⁻), intermediate products including ammonium (NH₄⁺), nitrite (NO₂⁻), and molecular nitrogen (N₂) are formed. In this reductive process, nitrate is initially converted to nitrite through reactions with hydrated electrons (e ⁻ _aq_) and hydrogen radicals (H^•^), followed by further reduction to nitrogen gas (N₂). Concurrently, variations in pH are observed due to the generation and consumption of protons during the reaction sequence [32]. The evolution of nitrate (NO₃⁻), ammonium (NH₄⁺), nitrite (NO₂⁻), nitrogen gas (N₂), and pH was monitored over 180 minutes under initial conditions of 50 mg/L nitrate, 150 mg/L sulfite, and pH 7. As illustrated in Fig 5, the concentration of nitrate decreased progressively, while nitrite levels initially rose to 9.6 mg/L at 30 minutes before declining to 0.1 mg/L by 180 minutes. A small amount of ammonium (0.05 mg/L) was also detected as a byproduct.

During the early stages of the reaction, the generation of hydrated electrons (e ⁻ _aq_) and hydrogen radicals (H^•^) led to enhanced reduction conditions, causing acidification of the medium. However, after approximately 60 minutes, continued reactions between the electrons, hydrogen radicals, and remaining pollutants induced an increase in pH, eventually restoring it close to its original value. These findings suggest that pH dynamics play a critical role in the reduction of nitrate within the SMP process.

The main mechanism of nitrate reduction is based on the reaction [15] and [16,32]. That nitrate is reduced in two stages. First, nitrate reacts with e_aq_^-^ and produces N_2_ [43,51].


$${{\text{NO}}_{\text{3}}}^{-}\;+\text{5}\;{{\text{e}}^{-}}_{\text{aq}}+\text{6}{\text{H}}^{+}\;\to \;\frac{\text{1}}{\text{2}}{\text{N}}_{\text{2}}\;(\text{g})\;+\;{\text{3H}}_{\text{2}}\text{O}$$ (16)

$${{\text{NO}}_{\text{3}}}^{-}\;+\text{2}\;{{\text{e}}^{-}}_{\text{aq}}+\text{2}{\text{H}}^{+}\;\to \;{{\text{NO}}_{\text{2}}}^{-}\;+\;{\text{H}}_{\text{2}}\text{O}$$ (17)

This reaction consists of two semi-reactions: a) semi-reactions converting NO_3_^-^ to nitrite


$${{\text{NO}}_{\text{2}}}^{-}\;+\text{3}\;{{\text{e}}^{-}}_{\text{aq}}+\text{4}{\text{H}}^{+}\;\to \;\frac{\text{1}}{\text{2}}{\text{N}}_{\text{2}}\;(\text{g})\;+\;{\text{2H}}_{\text{2}}\text{O}$$ (18)

b) semi-reaction of conversion of nitrite to nitrogen gas [52].


$${{\text{NO}}_{\text{3}}}^{-}\;+\text{8}\;{{\text{e}}^{-}}_{\text{aq}}+\text{10}{\text{H}}^{+}\;\to \;{{\text{NH}}_{\text{4}}}^{+}\;+\;{\text{3H}}_{\text{2}}\text{O}$$ (19)

As indicated in the reaction [19] NO_3_^-^ may become ammonium under acidic conditions [53].


$${{\text{NO}}_{\text{3}}}^{-}\;+\text{3}{\text{H}}^{\cdot }{\;+\;\text{H}}^{+}\;\to \;\text{NO}\;(\text{g})\;+\;{\text{2H}}_{\text{2}}\text{O}$$ (20)

Another mechanism has been proposed to reduce NO_3_^-^. NO_3_^-^ may react with H^•^ radicals and be restored to NO [54]. In S2 Fig in (S1 File), the schematic illustrates the reduction of nitrat.

To better illustrate the relative performance of the SMP process, a comparative summary of SMP and other reported treatment technologies for triclosan is presented in Table 3.

**Table 3:** Comparison of different technologies for triclosan removal.

Process	Mechanism	Removal rate	References
**Chlorination**	Degradation by the generated •OH	>92% TCS removal	[5]
**hydrated electron reduction**	Degradation by the e^-^_aq_	TCS removal ≈70	[55]
**Electrochemical oxidation**	Oxidative degradation UsingPd/nFe	>80% TCS removal; > 60% TOC removal	[5]
**Adsorption**	Chemisorption and physisorption	TCS removal (≈85–95%)	[4]
**SMP**	Degradation by the generated e^-^_aq_	Removal efficiencies (≈100%)	The present study

### 3.6. Percentage of selectivity of ammonium, nitrite, nitrogen

The results presented in S3 Fig in (S1 File) illustrate the selectivity percentages for ammonium (NH₄⁺), nitrite (NO₂⁻), and nitrogen gas (N₂) following treatment with an initial nitrate concentration of 50 mg/L, a sulfite concentration of 150 mg/L, and an initial pH of 7 over 180 minutes. Under optimal conditions, the selectivity toward N₂ was 97%, while the selectivity for ammonium and nitrite were 0.86% and 0.20%, respectively. These findings indicate that approximately 98% of the initial nitrate was successfully reduced and predominantly converted into nitrogen gas through the SMP process, demonstrating its high efficiency in achieving complete denitrification. In a study designed to reduce nitrate (100 mg/L) by photocatalysis with Ag2O/P25 at a concentration of 12.5%, an N2 selectivity of 82.9% was obtained after an irradiation time of 4 hours [56].

### 3.7. Investigating the effect of intervening factors on the NO_3_^-^ and TCS reduction

**Fig 6:**
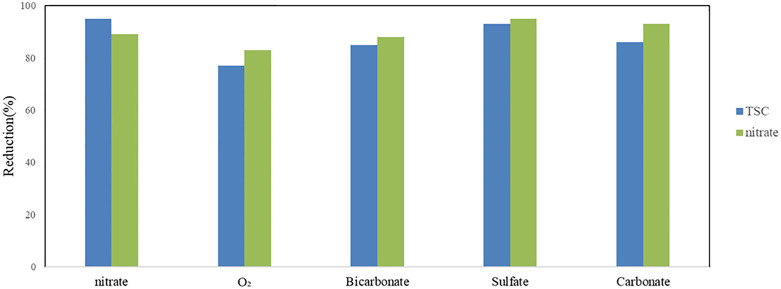
Effect of dissolved oxygen (O_2_) and inorganic anions (bicarbonate, carbonate, sulfate, and chloride) on the simultaneous reduction of nitrate and TCS (pH  = 7.0, time = 90 min, TCS = 50 mg L ^-1^, nitrate = 50 mg L ^-1^, sulfite = 150 mg L ^-1^).

Fig 6 illustrates the impact of dissolved oxygen and various anions, including bicarbonate, chloride, carbonate, and sulfate (each at a concentration of 50 mg/L), on the simultaneous reduction of nitrate and triclosan (TCS) under the conditions of pH 7.0, 90 minutes reaction time, 50 mg/L initial concentrations of TCS and nitrate, and 150 mg/L sulfite concentration. The degradation efficiencies of TCS in the presence of bicarbonate, carbonate, sulfate, chloride, and oxygen were 85%, 86%, 93%, 95%, and 77%, respectively. Correspondingly, nitrate reduction efficiencies under these conditions were 88%, 93%, 95%, 97%, and 83%. While the presence of anions exhibited negligible influence on the reaction, dissolved oxygen significantly decreased the removal efficiency. These findings suggest that hydrated electrons (e ⁻ _aq_) play a critical role in the degradation of TCS, which is consistent with the results reported by Yazdanbakhsh et al [57]. Among the investigated anions, bicarbonate exhibited the most pronounced inhibitory effect on TCS degradation, resulting in approximately a 15% reduction in removal efficiency. Bicarbonate was found to negatively influence the process by scavenging reactive radicals essential for TCS decomposition. In contrast, sulfate anions demonstrated the least inhibitory impact on the system’s performance. The minimal effect of sulfate is attributed to its dual role: while sulfate can absorb ultraviolet radiation, potentially diminishing light penetration, it also enhances the photochemical process by facilitating the conversion of sulfite to sulfite radicals (SO₃^•⁻^) and contributing to the direct degradation of TCS via sulfate radicals (SO₃^•⁻^) [58]. Moussavi and at.al showed that acetaminophen oxidation and nitrate reduction in the presence of all tested anions was > 96%. the main anions of water, including chloride, carbonate, and bicarbonate, have no significant effect on the performance of the VUV photoreactor [32].

### 3.8. TCS Dechlorination efficiency

Chlorine is a dangerous and persistent toxic compound in the structure of TCS. Therefore, It is necessary to separate chlorine from the structure of chlorinated benzene compounds. Therefore. As shown in S4 Fig in (S1 File), the amount of chlorine released into the solution during the simultaneous reduction of nitrate and TCS under the conditions of pH 7.0, a reaction time of 180 minutes, 50 mg/L TCS, and 150 mg/L sulfite is depicted.. The findings are shown, As the decomposition time of TCS increases, more chlorine is released from TCS into the solution.

### 3.9. TCS mineralization rate evaluated by COD and TOC indices

The chemical oxygen demand (COD) and total organic carbon (TOC) indices were employed to assess the mineralization of effluent from the SMP process, with an initial concentration of 50 mg/L of triclosan (TCS). After 90 minutes of reaction, the COD and TOC removal efficiencies were observed to be 38% and 16%, respectively (S5 Fig in S1 File). The observed mineralization in the SMP process can primarily be attributed to the generation of reactive radical species—especially sulfite radicals (SO₃^•⁻^), hydrated electrons (e ⁻ ₐq), and hydrogen radicals (H^•^)—which are known to catalyze the extensive degradation of organic pollutants. These species not only facilitate the breakdown of complex molecular structures but also contribute to the conversion of intermediate organic fragments into simpler, more stable inorganic compounds, ultimately leading to carbon dioxide (CO₂) and water (H₂O).

Although the SMP process is categorized under advanced reduction mechanisms, the presence of dissolved oxygen (DO ≈ 2 mg/L) in the reaction system may contribute in a secondary role, especially in the further oxidation of partially degraded byproducts. UV activation of sulfite, in particular, can indirectly generate oxidative species, especially in the presence of trace amounts of oxygen, promoting mineralization through a synergistic mechanism.However, the primary driver of mineralization appears to be radical-induced degradation rather than oxygen-facilitated oxidation, as indicated by the substantial reductions in both TOC (16%) and COD (30%) under low-oxygen conditions. Further studies under controlled oxygen environments may help elucidate the role of O₂ in enhancing or limiting the efficiency of the mineralization process. In the study by Asgari et al., on the mineralization and biodegradability improvement of textile wastewater using persulfate/dithionite process, the results showed that the COD and TOC removal efficiency reached 64.9 and 54.5%, respectively, but it did not meet the required discharge conditions and standards [59].

### 3.10. Electrical energy per order(EEO)

Eq. (14) is the total cost of the system (TCS) ($ m^-3^). in which unit is EEM kWh m^3^, $/kWh Power cost, and reductant cost is $g^-1^. As Table 4 shows, the lowest values of E_EM_ and TCS were for SMP process, considering the low values of E_EM_ and TCS for SMP, this process can be suitable and economical. The alternative is the simultaneous removal of triclosan and soluble nitrate. Although SMP involves the generation of radical species such as hydrated electrons (e ⁻ ₐq) and sulfite radicals (SO₃^•⁻^), its operational cost is lower than conventional AOPs/ARPs. As shown in Table 4, SMP required less energy and showed higher efficiency under neutral pH. In addition, the process avoids using harmful reagents like ozone or hydrogen peroxide, making it safer and more environmentally friendly. In the study by Yazdanbakhsh et al.[60], the electrical energy required to decompose sulfamethoxazole per cubic meter of solution ranged from 2.2 to 21.92 kWh/m^3^.

**Table 4:** Energy consumption and kinetic parameters of different processes used in TCS and NO₃ ⁻ removal.

Process	Kobs(min-1)		EEM (kWh m-3)		Total cost ($ m-3)	
	NO3-	TCS	NO3-	TCS	NO3-	TCS
alone UV	0.001	0.001	NO3-	2.6	0.46	0.41
Sulfite alone	0.004	0.022	0.53	4.26	-	-
SMP	0.017	0.031	4.1	0.2	0.39	0.38
			0.3			

### 3.11. Reaction pathways and intermediates of TCS destruction by radical

To study the final products, the identifying intermediate formed upon photochemical degradation, TCS decay (with a maximum concentration of 250 mg L^-1^) was done by the SMP process at pH = 7, and a time of 30 min. Supplementary S6 Fig in S1 File shows the results of the LC/MS analysis of the final products in the SMP process in a negative state by [M-H] and in the range of 5/0–400 m/Z. The Proposed major intermediate species and structures are shown in Supplementary S1 Table in S1 File (under necessary conditions). that TCS was not detected in the final by-products, indicating that the process successfully dechlorinated the compound. The chlorine-containing species that formed during the degradation process were low-intensity intermediates, further confirming that the carbon-chlorine bond in TCS was effectively cleaved. Which shows Radical sulfite has a significant effect on TCS chlorination based on LC-MS analysis. According to LC-MS data analysis in Supplementary S7 Fig in S1 File the intermediate limits formed by this degradation include 4,5’-dichloro-[1,1’-biphenyl]-2,2’-diol (m/z = 255), 2,8-dichlorodibenzo[b, e][1,4]dioxine (m/z = 253), 2-chlorodibenzo[b, e][1,4]dioxine (M/Z = 218), 2-phenoxy phenol (M/Z = 186), 2,5-dichlorophenol (m/z = 161), 4-chlorophenol (M/Z = 129) and other structures and molecules with less molecular masses. HPLC Spectra indicate that the material is pure. The identified intermediates (S1 Table in S1 File and the proposed pathway in S7 Fig in S1 File) are consistent with initial dechlorination and subsequent cleavage of the phenyl-ether/phenolic moieties of TCS. Although compound-specific concentration and toxicity data for these intermediates were not obtained, whole-effluent acute bioassays using Daphnia magna show a marked decrease in toxicity following SMP treatment (TU48 decreased from 15.22 to 1.48; Table 5), indicating an overall reduction in acute toxicity at the effluent level. Therefore, under the tested conditions there is no evidence that the SMP process produces more toxic contaminants than the parent compound.

**Table 5:** Changes in effluent toxicity at different times of the process.

Solution	Time (h)	LC_50_ (v.v)%	Confidence Limits 95%		Toxicity Unit TU
			Higher limitLower limit		
	24	13.30%	21.9%	7.30%	7.51
Raw waste water	48	6.57%	57%	3.7%	15.22
	72	2.4%	4.4%	1.4%	40.60
	96	1.3%	2.3%	0.53%	76.9
	24	30.39%	4603.60%	20.9%	3.29
After treatment	48	67.5%	266.57%	66.7%	1.48
	72	92.4%	109.4%	48.4%	1.08
	96	110%	177.6%	21.3%	0.9

### 3.12. Toxicity assessment

Table 5 shows the results of the number of deaths of *Daphnia magna* in the effect of exposure to different dilutions of raw sewage and filtered sewage. The results show that the percentage of Daphnia mortality increases with time during 24, 48, 72 and 96 hours. According to the Table 5 values of LC_50_, 24, 48, 72, and 96 hours at the end of the refining time are equivalent to 30.39,67.5, 92.4, and 110 percent respectively (v: v) And the 48-h toxicity unit (TU) also went from the initial value of 15.22 units at the beginning of the process to 1.48 units at the end of the process, with the equivalent of 10.28 times the reduction in toxicity. According to the classification, the toxicity of toxic sewage was reported by the TU_48_-H toxicity unit as zero (non-toxic) ranges, 1 > slightly toxic, 1–10 toxic, 10–100 highly toxic, and 100 > infinitely toxic. The results show that the 96-h toxicity was equal to 76.6, which decreased to 2.94 after the SMP process. This decreasing trend was observed at all times of 24, 48 and 72 hours.

### 3.13. Investigating the type of simulated water type

The SMP process affects the quality of water, and its type. Because the anions and cavities in the water are important indicators of the type and face of the water, the presence of water anions showed a small impact on the functioning of the reduction process [61]. The negative effect on process function is observed when anions react with eaq and thus reduce the amount of eaq in the solution, which results in the production of anionic radicals that are considered weak compared to eaq reagents Sulfate, NO_3_^-^ and carbonate are the complete oxidation of sulfur, nitrogen and carbon elements. These anions tend to regenerate rather than oxidize. They react with hydrated electrons in the solution, but due to the low-importance role of electrons in the reduction of contaminants from aqueous solution, the presence of these anions cannot affect the regeneration of contaminants. Bicarbonate and chloride anions also tend to lose electrons instead of taking them. However the results clearly show that water anions cannot stop the efficiency of radicals [32,37]. The results showed that before the process, the type of water was bicarbonate-calcium and after the process it was also bicarbonate-calcium (HCO_3_-Ca).

Taken together, our results show that the UV/sulfite/phenol sulfite-mediated photoreduction (SMP) system performs strongly for the concurrent abatement of co-occurring organic and inorganic contaminants in water. Increasing the initial pollutant loading enhances the photogeneration of reactive reducing species—chiefly hydrated electrons (eaq^−^) and sulfite radical anions (SO3^•−^)—and thus expands the system’s reductive capacity. Nevertheless, the *fractional* (percent) removal declines at higher influent concentrations, most likely because radical demand outstrips in situ production and because light screening, competitive scavenging, and accumulation of transformation products consume the available reactive species. Collectively, these findings identify advanced reduction processes (ARPs) as promising technologies for treating wastewaters that contain elevated levels of recalcitrant or toxic compounds. Given that many antibiotics and other pharmaceutically active compounds incorporate halogenated or otherwise electron-deficient aromatic moieties analogous to triclosan, we recommend targeted future investigations into the reductive transformation of representative antibiotics under optimized ARP conditions. Overall, the SMP process shows a synergistic effect between UV irradiation and sulfite activation, leading to efficient removal of inorganic (NO₃⁻) and organic (TCS) pollutants under mild conditions. The findings highlight the dual reduction-oxidation capacity of the system, which contributes to the detoxification and mineralization of pollutants with minimal energy input.

### 3.14. Future Perspectives and Practical Implementation

Future research should aim to advance the scalability and real-world applicability of the SMP process beyond laboratory-scale experiments. In particular, its performance should be rigorously assessed under complex wastewater matrices, where variable chemical compositions and interfering constituents could influence treatment outcomes. Investigations into the system’s operational robustness and long-term stability during continuous flow conditions are also critical to determine its viability for sustained application.

To support scale-up, pilot- or semi-industrial-scale reactor studies are essential for evaluating hydraulic dynamics, energy consumption, and cost-efficiency under realistic operational parameters. Complementary to these efforts, simulation tools—such as kinetic modeling and computational fluid dynamics (CFD)—can provide valuable insights for optimizing process conditions and reactor design in large-scale implementations.

Importantly, the SMP process should not be viewed as a replacement for existing treatment technologies, but rather as a complementary strategy. When integrated with conventional biological treatment systems, SMP can serve as a post-treatment or polishing step, effectively targeting recalcitrant organic compounds and halogenated byproducts. Such hybrid configurations may improve overall treatment performance while reducing residual toxicity and mitigating ecological risks.

## 4. Conclusion

The main objectives of this study were to evaluate the effectiveness of the SMP (sulfite-phenol) process for the simultaneous removal of triclosan and nitrate in aqueous solutions, dechlorination, mineralization and bioassay of TCS with Daphnia magna and the percentage of nitrite, ammonium and N2 selectivity, energy efficiency and toxicity assessment of the produced water. This process can destroy resistant halogenated compounds such as TCS by breaking C-Cl bonds in chlorinated organic compounds and also regenerate the most stable nitrogen state by generating non-selective and highly reactive radicals such as hydrated electrons (eaq-) and hydrogen radicals (H^•^).The highest removal efficiencies occurred at neutral pH, with enhanced performance observed at increasing sulfite concentrations. While UV or sulfite alone showed minimal activity, their combination with phenol led to 100% TCS degradation and 68% nitrate reduction. Although SMP improved mineralization, complete mineral breakdown required extended irradiation. Mass spectrometry revealed no residual TCS, with low-intensity chlorine-containing peaks indicating effective cleavage by sulfite radicals. Toxicity assays confirmed a substantial reduction in acute toxicity (from 76.6 to 2.94 TU), and the water type (HCO₃–Ca) remained unchanged post-treatment. Since the toxicity and stability of chlorinated compounds are closely related to the C-Cl bond, reductive degradation is easily achieved. Preservation of the carbon skeleton containing the hydroxyl radical by completely covering it with electronegative chlorine substituents, contrary to the electronegative strength of chlorine atoms, can act as a reducing reaction center for dechlorination, so the SMP system is a promising and sustainable approach, emphasizing its application value and long-term potential. for the removal of organic and nitrogenous pollutants from wastewater. Sulfite acts as an efficient reducing agent to break the carbon-halogen bonds in halogenated organic pollutants. Our findings show that although this process achieves a high degree of dechlorination, its limited mineralization capacity suggests that it is best performed as a final step after biological processing. In future studies, it is proposed to use biological systems and microorganisms capable of producing electrons for the advanced reduction of halogenated organic compounds at a lower cost and in an environmentally friendly manners. This approach has significant long-term potential for sustainable environmental remediation and industrial wastewater treatment, offering a promising solution to the challenges posed by persistent organic pollutants. For completeness, future work could include targeted quantification of key intermediates and compound-specific toxicity testing (or QSAR/fractionation approaches) to better resolve the hazard potential of individual transformation products.

## Supporting information

S1 File Supplementary figures and tables. This file contains Figures S1–S7 and Table S1 that support the findings reported in the main text.
(DOCX)
