# Supplementary material for: Simultaneous triclosan degradation and nitrate reduction by a UV/Sulfite/Phenol process based on sulfite radical mechanism: dechlorination, mineralization, and bioassessment
Source: PLoS One. 2026 Feb 6;21(2):e0340396. doi: 10.1371/journal.pone.0340396 (PMC12880720; doi:10.1371/journal.pone.0340396)
Supplement: S1 File — This file contains Figures S1–S7 and Table S1 that support the findings reported in the main text. (DOCX) [file pone.0340396.s001.docx]

Fig. S1. Comparison of the efficiency of UV,Sulfite and SMP processes (pH= 7.0, Time, 90 min, TCS = 50 mg L^-1^and nitrate = 50 mg L^-1^).


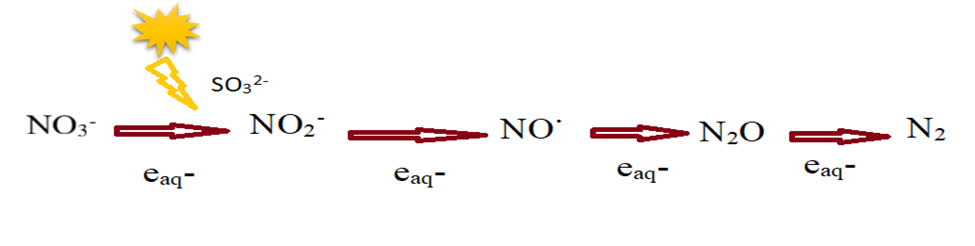


Fig. S2. the schematic of the reduction of nitrat

Fig. S3. Percentage of selectivity of ammonium, nitrite, N_2_

Fig. S4.The amount of free and combined chlorine in solution.

Fig. S5. The rate of TCS degradation, COD and TOC Reduction (pH, 7.0; TCS = 50 mg L^-1^; Sulfite=100 mg L^-1^).


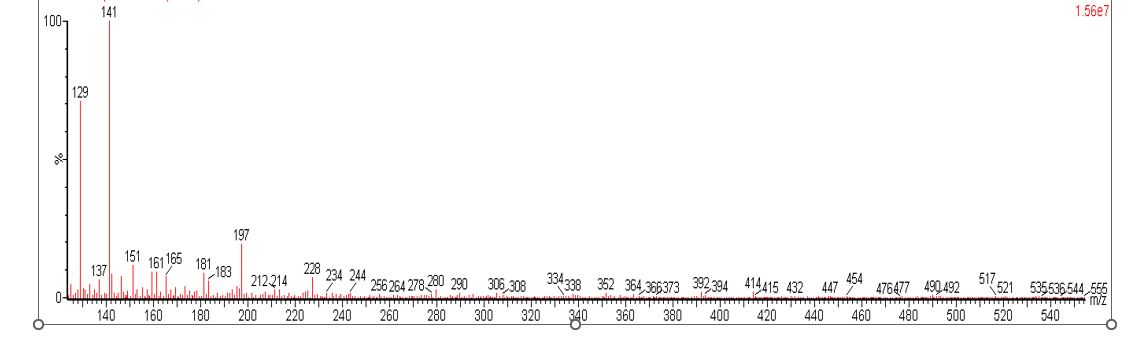


Fig. S6. LC/MS ion chromatograms of the TCS solution-treated SMP process.

**

Fig. S7. LC/MS ion chromatograms (a) and proposed reaction pathway (b) for the TCS solution treated by SMP process

Tables

Table S1. Intermediate compounds produced from the decomposition of TCS

| No. | Structure | Name | Chemical formula | Detected mass  (m/z) |
| --- | --- | --- | --- | --- |
| 1 |  | 5-chloro-2-(2,4-dichlorophenoxy) phenol | C_12­_H_7_Cl_3_O_2_ | 290 |
| 2 |  | 4,5'-dichloro-[1,1'-biphenyl]-2,2'-diol | C_12­_H_8_Cl_2_O_2_ | 255 |
| 3 |  | 5-chloro-2-(4-chlorophenoxy) phenol | C_12­_H_8_Cl_2_O_2_ | 255 |
| 4 |  | 2,8-dichlorodibenzo[b,e][1,4]dioxine | C_12­_H_6_Cl_2_O_2_ | 253 |
| 5 |  | 2-chlorodibenzo[b,e][1,4]dioxine | C_12­_H_7_ClO_2_ | 218 |
| 6 |  | 4-chloro-[1,1'-biphenyl]-2,2'-diol | C_12­_H_9_ClO_2_ | 220 |
| 7 |  | 5-chloro-2-phenoxyphenol | C_12­_H_8_ClO_2_ | 220 |
| 8 |  | [1,1'-biphenyl]-2,2'-diol | C_12­_H_10_O_2_ | 186 |
| 9 |  | 2-phenoxyphenol | C_12­_H_10_O_2_ | 186 |
| 10 |  | 2,5-dichlorophenol | C_6­_H_4_Cl_2_O | 161 |
| 11 |  | 4-chlorobenzene-1,2-diol | C_6­_H_5_ClO_2_ | 141 |
| 12 |  | 4-chlorophenol | C_6­_H_5_ClO | 129 |
